# Characterization of atopic dermatitis in older adults: A retrospective study

**DOI:** 10.1016/j.jdin.2024.03.028

**Published:** 2024-04-30

**Authors:** Hannah Y. Wang, Denise C. Robson, Joanne S. Jacob, Milbrey A. Parke, Louisa Y. Liu, Soo Jung Kim

**Affiliations:** a School of Medicine, Baylor College of Medicine, Houston, Texas; b Department of Dermatology, The University of Alabama at Birmingham, Birmingham, Alabama; c Department of Dermatology, University of Florida College of Medicine, Gainesville, Florida; d Department of Dermatology, Loma Linda University Health, Loma Linda, California; e Department of Dermatology, Baylor College of Medicine, Houston, Texas

**Keywords:** atopic dermatitis, eczema, elderly

*To the Editor:* Atopic dermatitis (AD) is an inflammatory skin condition that commonly presents in childhood. However, 2% to 10% of adults are affected by AD, either from recurrent childhood disease or new-onset disease in their adulthood.^1^ Patients ≥60 years of age have been shown to present with unique features, prompting us to further examine the characteristics of AD in elderly patients.^2^

We performed a cross-sectional study and reviewed charts of patients ≥60 years of age seen at either a private or county dermatology clinic in Houston, Texas. Records were searched from 2009 to 2020 and included if the patient had a diagnosis of AD by a dermatologist designated by the International Classification of Diseases code. Clinical data such as patient demographics, rash characteristics, treatments, and atopic history were compiled.

We identified 791 patients who met the inclusion criteria, 476 (60.1%) of whom were female (Table I). Prior studies demonstrate mixed results with regards to whether a sex predominance exists for AD in elderly patients.^2^^,^^3^ The average age of these patients was 69.3 years, and 315 (39.6%) of whom were non-Hispanic White. Lichenification (14.5%) and nummular lesions (12.8%) were the most frequent rash characteristics. Lichenification seems to be more prevalent in in adults with AD compared to children with AD, which may reflect prolonged and recalcitrant disease activity in adults.^4^ The most common locations for AD lesions in these elderly patients was on the extensor surfaces (49.9%) and trunk (46.0%), and the least common locations were on the hands (19.8%) and feet (9.0%). These findings share overlaps with a study by Silverberg et al that found the trunk (67.4%) as the most common location for AD lesions in American patients ≥60 years, with a similar frequency of flexural surface involvement (39.6% vs 34.9%).^5^ A study on Japanese patients by Tanei et al also found that lesions on the upper extremities (95.0%) and trunk (91.7%) were seen in almost all adults ≥60 years with AD.^2^

**Table I:** Demographics and disease characteristics of elderly patients with atopic dermatitis

	Total (*N* = 791)
Age (y)	
Mean (SD)	69.3 (7.5)
Median (IQR)	68 (63-73)
Sex	
Male	315 (39.8)
Female	476 (60.2)
Race	
Non-Hispanic White	314 (39.6)
Non-Hispanic Black	173 (21.8)
Hispanic	162 (20.4)
Non-Hispanic Asian/Pacific Islander	93 (11.7)
Other/unknown	49 (6.2)
Rash characteristics	
Nummular	101 (12.7)
Ichthyosis	77 (9.7)
Hyperpigmented patches	72 (9.1)
Lichenified	115 (14.5)
Distribution	
Flexors	314 (39.6)
Extensors	395 (49.9)
Face/neck	210 (26.5)
Trunk	364 (46.0)
Hands	157 (19.8)
Feet	71 (9.0)
Prescribed medications	
All topicals	741 (93.6)
Topical corticosteroids	730 (92.2)
Topical nonsteroidal∗	60 (7.6)
Oral corticosteroids	83 (10.4)
Antihistamines	96 (12.1)
Dupilumab	43 (5.4)
Systemic immunosuppressants†	43 (5.4)
Apremilast	4 (0.5)
UV-B–phototherapy	21 (2.7)
Atopic triad history	
Asthma	156 (19.7)
Allergic rhinitis	315 (39.8)

∗ Topical nonsteroidal medications included pimecrolimus, tacrolimus, and crisaborole.

† Systemic immunosuppressants included methotrexate (26), cyclosporine (22), and mycophenolate (2). Some patients took more than 1 systemic immunosuppressant.

Oral corticosteroids (10.4%) and antihistamines (12.1%) were frequent systemic treatments, though this is likely due to management prior to a diagnosis of AD by a dermatologist; 39.8% of patients also had a history of allergic rhinitis, while a history of asthma was less common (19.7%). This study further supports that AD in elderly patients presents as a unique phenotype compared to AD in younger ages, which may inform dermatologists’ diagnosis of AD in these patients. The study benefited from a large sample of elderly patients with AD with racial and clinic type diversity as well as treatments tried. However, the accuracy and completeness of the data were dependent on the patient encounter documentation quality. Age-related factors that may impact the presentation of AD in elderly patients include reduced skin barrier function, immune dysregulation, and environmental exposures.^1^ However, further investigations are needed to elucidate the unique features of elderly AD in pathophysiology and optimal treatments.

## Conflicts of interest

None disclosed.
